# Growth factors, body composition and energy expenditure in late preterm and term infants during the first 4 months of life: a prospective cohort study

**DOI:** 10.1186/s40348-025-00201-4

**Published:** 2025-10-01

**Authors:** Niels Rochow, Anna-Lisa V. Nguyen, Gerhard Fusch, Gisela Adrienne Weiss, Hon Yiu So, Hansjörg Rudolf Schmelzle, Christoph Fusch

**Affiliations:** 1 Department of Pediatrics, McMaster University, Hamilton Health Sciences, Hamilton, ON L8S 4L8 Canada; 2 Department of Pediatrics, Paracelsus Medical University, Breslauer Str. 201, Nuremberg, 90471 Germany; 3 Department of Pediatrics, Greifswald University Medical Center, Greifswald, 17475 Germany; 4 Department of Pediatrics, Rostock University Medical Center, Rostock, 18057 Germany; 5 Schulich School of Medicine and Dentistry, Western University, London, ON N6A 5C1 Canada; 6 Department of Health Research Methods, Evidence and Impact, McMaster University, Hamilton, ON L8S 4L8 Canada; 7 Department of Mathematics and Statistics, Oakland University, Rochester, MI 48309 USA; 8 Fachklinik Caritas-Haus Feldberg, Feldberg, 79868 Germany

**Keywords:** Growth, Hormones, Biomarkers, Insulin-like growth factor, Leptin, Fat mass, Fat-free mass, Weight, Length, Postnatal fat accretion

## Abstract

**Background:**

Late preterm infants (34–36 weeks gestation) represent the majority of preterm births and are often assumed to follow similar postnatal growth trajectories as term infants. However, the postnatal hormonal environment and body composition development in this group remain underexplored. This prospective observational study aimed to analyze and compare growth, body composition, energy expenditure, hormonal, and metabolic responses in healthy late preterm and term infants in the first four months of life.

**Results:**

Anthropometry, body composition, energy expenditure, metabolic biomarkers and growth factors were measured in 94 term infants (gestational age: 39.6 ± 1.3 weeks, birth weight 3330 ± 570 g) and 18 late preterm infants (35.0 ± 1.0 weeks, 2520 ± 660 g) at three time points (0–5, 55–65 and 115–125 days of life). The onset of fat mass accretion occurred directly after birth resulting in higher percent fat mass in late preterm infants in early life. Late preterm infants reached a similar percent fat mass approximately five weeks earlier in postmenstrual age than term infants. In contrast, fat-free mass developed along similar trajectories in both groups, indicating preserved lean tissue growth in late preterm infants. Energy expenditure doubled during the first two months and was closely linked to fat-free mass accretion. Insulin-like growth factor (IGF)-1 and IGF-2 levels increased postnatally, with slightly higher concentrations in late preterm infants. Increase of percent fat mass paralleled leptin and IGF levels in both groups. IGF-1 and IGF-2 levels were higher in formula-fed infants, supporting the influence of nutritional composition on growth-related hormonal regulation.

**Conclusions:**

Birth may initiate changes in hormonal levels and acceleration of fat mass accrual, resulting in higher fat mass in late preterm-born infants at term age when compared to term-born infants. Next to hormonal shifts, these changes appear to be driven by nutritional factors in the early postnatal period. The results suggest that growth targets for late preterm infants may need to be reconsidered, particularly in the early postnatal period. Future studies should provide evidence on individual growth targets and nutritional guidelines for preterm infants to account for the physiological differences to term infants.

## Impact

• This study describes the interplay of growth, body composition, hormones, energy expenditure, and metabolic parameters in healthy term-born and late preterm infants.

• At term age, fat mass in late preterm infants was higher while fat-free mass was similar when compared with term infants.

• Results suggest that birth initiates postnatal adaptation and hormonal changes triggering the acceleration of fat mass accrual, thereby setting off the deviation from fetal growth and body composition references.

• The observed early accrual of fat mass initiated by birth suggests different weight targets for late preterm infants at term age when compared with term infants.

## Background

Current research in neonatal intensive care focuses on improving quality of life. Lifelong health including cardiovascular and metabolic diseases in adulthood has been shown to be related to nutrition, growth and body composition during the first year of life [1–5].

Nutritional committees propose that a preterm-born infant should develop similarly to a healthy fetus that stays in utero until term age. Hence, preterm-born infants are expected to mimic the growth, body composition, and neurodevelopment of term-born counterparts [6]. However, it has been shown that preterm infants with low gestational age – even when reaching similar weights as term infants – are shorter and have higher fat mass and reduced fat-free mass [7–9].

**Fig. 1:**
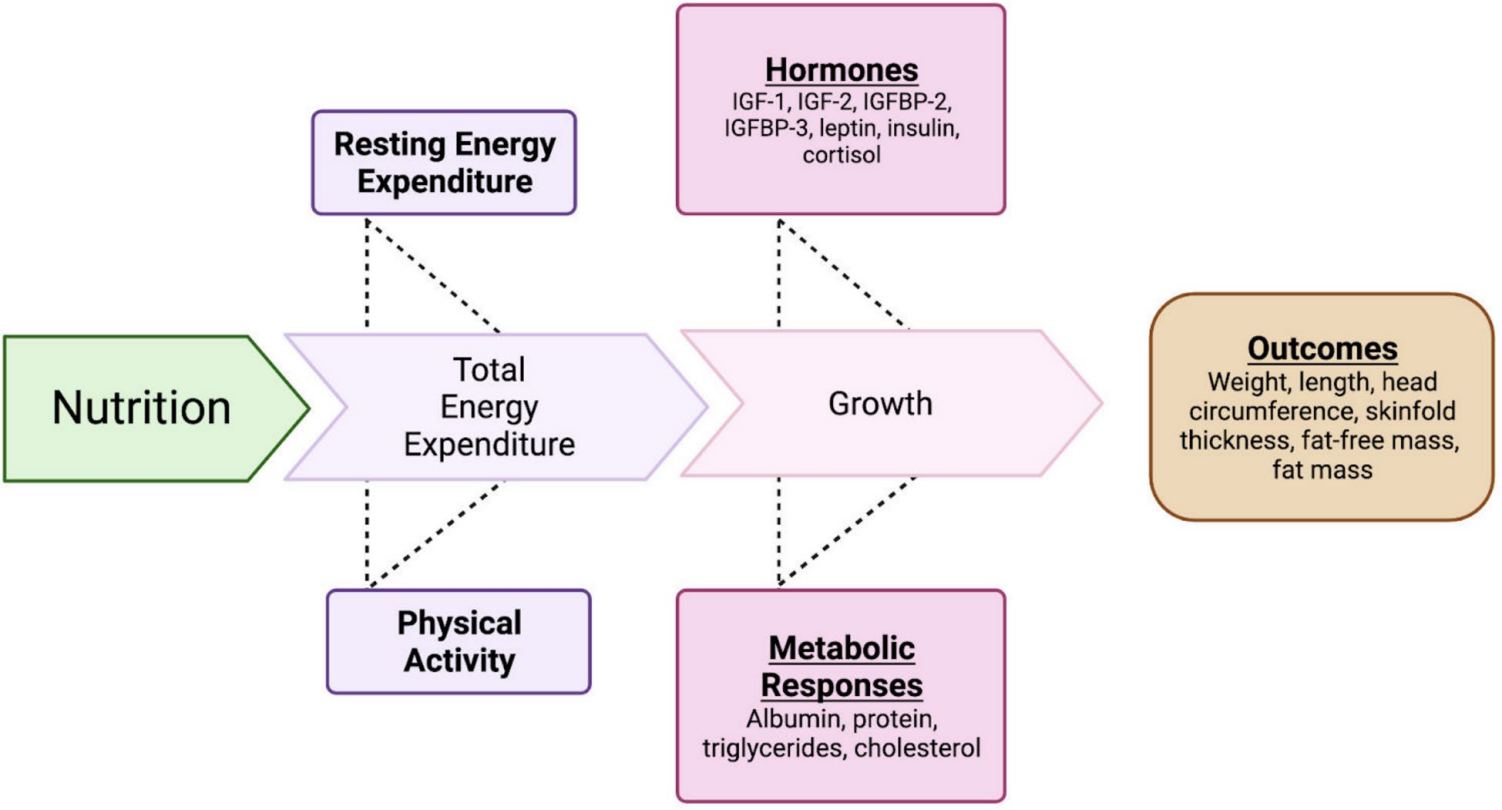
Conceptual model linking nutritional intake, energy expenditure, biomarkers and growth in preterm and term-born infants [9, 10]. Parameters measured in this study include growth promoting hormones, metabolic parameters, anthropometric measurements and body composition were measured in this cohort. IGF, insulin-like growth factor, IGFBP, insulin-like growth factor binding protein

The hypothesis posited that early exposure to the extrauterine environment contributes to differences in growth and body composition between preterm and full-term infants [7–9]. The transition from intrauterine to postnatal life encompasses numerous physiological shifts, including the shift from placental to postnatal nutrition, cessation of materno-placental hormonal influences, adjustments in cardiorespiratory functions, and thermoregulation adaptations [7–9]. These changes are orchestrated by a multifaceted interplay of factors, as earlier proposed [10]. The interaction is characterized by nutritional input triggering metabolic responses, elevating energy expenditure, and causing changes in hormonal equilibrium. Consequently, escalating levels of growth-inducing hormones, like insulin-like growth factors (IGFs), insulin-like growth factor binding proteins (IGFBPs), and leptin, exert influence over metabolic parameters (e.g., protein, albumin, triglycerides). This interplay is thought to underpin the modulation of physiological postnatal growth and body composition, where a harmonious balance between these parameters is deemed essential (see Fig. 1). However, little information displaying the entirety of this interplay has been published in late preterm and term infants during the first months of life. Therefore, this observational study aims to analyze the growth, body composition, hormones, energy expenditure, and metabolic parameters in healthy term-born infants as a model for normal growth and to compare these parameters with late preterm infants.

## Methods

### Study design

This prospective observational cohort study was conducted at the level III neonatal intensive care unit of University Children’s Hospital of Greifswald, Germany. The study was approved by the research ethics board of the University of Greifswald (REB#: UV 26/98) and parental consent was obtained.

### Study subjects

Healthy infants born at 34 to 42 weeks of gestation were included during the first days of life (postpartum day 0 to 5). The gestational age was based on the first trimester ultrasound. All infants were fed ad libitum with breast milk or standard formula provided 3.7 g fat, 1.6 g protein, and 7.0 g carbohydrate per 100 mL. Exclusion criteria were major congenital and chromosomal anomalies, mechanical ventilation, uncertain gestational age, conditions which affect fetal and neonatal growth (e.g. maternal diabetes, abnormalities of the placenta, maternal use of drugs), metabolic syndromes, multiple births, arterial cord blood pH < 7 and 10 min Apgar score < 7.

### Time points for outcome measurements

**Fig. 2:**
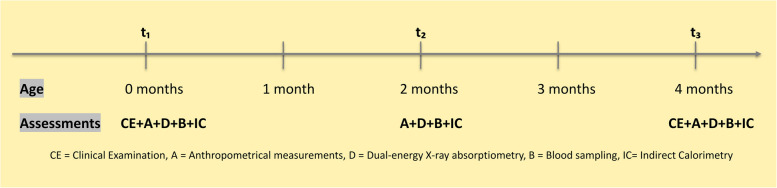
Overview of time points and measured outcome parameters

Outcome measurements for late preterm and term infants were performed at corresponding days of life. Time points at which measurements were taken included t_1_ = 0 to 5 days postpartum, t_2_ = 55 to 65 days postpartum (2 months), t_3_ = 115 to 125 days postpartum (4 months) (Fig. 2). Cord blood was collected at birth while venous blood was obtained at t_2_ and t_3_ prior to feedings.

### Anthropometry

Growth measurements were obtained by one assessor to reduce interobserver variation. We measured weight (duplicates, standard beam), supine length (triplicate, length board, Schäfer, Karlsruhe, Germany), head circumference (triplicate, non-stretchable standard tape), and skinfold thickness (triplicate, skinfold caliper, Holtain Ltd, Croswell, Crymych, United Kingdom) [11]. Percentiles were calculated based on the German national perinatal survey [12].

### Body composition parameters

Measurements for percent and absolute fat mass, fat-free mass, and bone mineral density were obtained by dual-energy X-ray absorptiometry using a whole-body densitometer (QDR-1500. Hologic, Waltham, MA, USA) [13]. All scans were analyzed with a modified infant whole-body software package (version 5.67) [14]. Fat-free mass index (fat-free mass/length^2^), and fat mass index (fat mass/length^2^) were calculated.

### Biomarkers

Plasma IGF-1 and IGF-2 levels were measured by an IGFBP-blocked radioimmunoassay (Mediagnost, Tübingen, Germany) [15]. IGFBP-3 and IGFBP-2 were measured using radioimmunoassays reported elsewhere [16]. Leptin was measured in duplicates using a radioimmunoassay kit (Mediagnost, Tübingen, Germany). Plasma albumin, protein, triglyceride, cholesterol, cortisol, and insulin levels were measured in the central lab of the University Hospital of Greifswald, Germany.

### Indirect calorimetry

Resting energy expenditure was assessed by indirect calorimetry using a Deltatrac II metabolic monitor (Datex, Finland). An ethanol burning test was performed to ensure proper calibration of the calorimeter [17].

### Data analysis

Descriptive statistics were employed for baseline and outcome measures. Data were presented according to postmenstrual age and chronological age. Mean and standard deviation were calculated for continuous variables and proportion was calculated for categorical variables. The association between outcome variables was tested using correlation analysis. Linear mixed models were employed to analyze longitudinal associations on growth hormones, and nutrition on body composition with repeated measurements. Regression coefficients, intercepts, and confidence intervals (CIs) were calculated. Different random intercepts for each infant were assumed. The level of significance was defined as *p* < 0.05. The statistical analysis was performed with R version 3.6.0 (2019–04-26) [18].

## Results

The study included 18 late preterm infants and 94 term infants. The two groups differed in gestational age by nearly five weeks, which corresponded to expected differences in birth weight, length, and head circumference. Despite these absolute differences, the percentiles for weight, length, and head circumference – adjusted for gestational age and sex – were not statistically different between late preterm and term infants. Detailed patient characteristics are presented in Table 1.

**Table 1:** Patient characteristics

	Late preterm infants	Term infants
n	18	94
Gestational age (weeks)	35.0 ± 1.0	39.6 ± 1.3
Birth weight (g)	2516 ± 663	3332 ± 571
Birth weight percentile	55 ± 32	43 ± 28
Birth length (cm)	46.8 ± 4.1	51.0 ± 2.5
Birth length percentile	64 ± 31	59 ± 25
Head circumference (cm)	32.5 ± 2.2	35.0 ± 2.2
Head circumference percentile	62 ± 29	56 ± 28
Sex (male/female) (n)	11/7	52/42
Breast milk/Formula (n)	3/15	56/38

Values are presented as means ± standard deviation, except for absolute numbers of male/female patients and patients receiving breast milk or formula

### Anthropometric and body composition measures

**Fig. 3:**
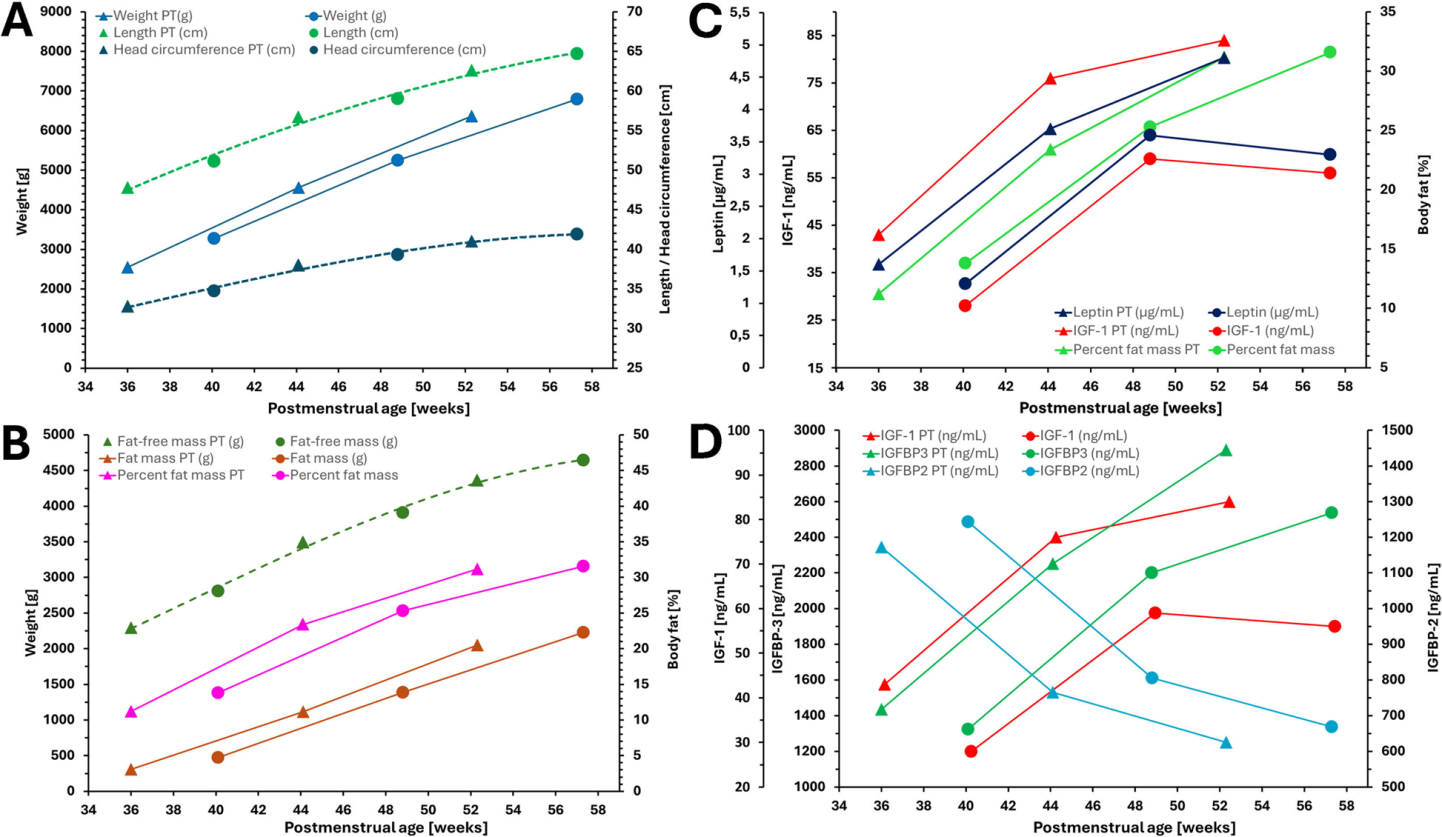
Trends in changes of hormones and growth. IGF, insulin-like growth factor; IGFBP, insulin-like growth factor binding protein; PT, preterm (triangles), term infants (circles)

The increase of length and head circumference followed the same trajectory when comparing preterm and term infants, while the weight trajectory of preterm infants was at a higher level (Fig. 3A, Table 2).

**Table 2:** Anthropometric measurements, body composition, results of indirect calorimetry, hormonal status and biomarkers of metabolism

Time points	t_1_		t_2_		t_3_	
**Group**	**late preterm**	**term**	**late preterm**	**term**	**late preterm**	**term**
Postmenstrual age	36.0 ± 0.8	40.1 ± 1.3	44.1 ± 1.1	48.8 ± 1.3	52.3 ± 1.3	57.3 ± 1.4
Day of life	9 ± 5	5 ± 3	66 ± 5	65 ± 6	123 ± 4	124 ± 5
**Anthropometry**						
n	18	94	12	70	12	58
Weight (g)	2549 ± 463	3271 ± 541	4560 ± 714	5252 ± 686	6363 ± 966	6798 ± 839
Body mass index (kg/m^2^)	11.1 ± 1.2	12.4 ± 1.2	14.1 ± 1.3	15.0 ± 1.2	16.2 ± 1.6	16.2 ± 1.5
Length (cm)	47.8 ± 2.5	51.1 ± 2.6	56.7 ± 2.8	59.0 ± 2.7	62.6 ± 3.0	64.7 ± 2.5
Head circumference (cm)	32.8 ± 1.4	34.7 ± 1.3	38.0 ± 1.2	39.3 ± 1.1	41.0 ± 1.2	41.9 ± 1.0
Average skinfold thickness	3.9 ± 0.7	4.0 ± 0.7	5.9 ± 1.3	6.4 ± 1.2	7.7 ± 1.4	7.1 ± 1.2
**Body composition**						
Percent fat mass	11.2 ± 4.0	13.8 ± 4.1	23.4 ± 7.3	25.3 ± 6.0	31.2 ± 6.5	31.6 ± 6.7
Fat mass (g)	309 ± 148	476 ± 201	1115 ± 452	1386 ± 464	2053 ± 590	2229 ± 623
Fat mass index (kg/m^2^)	1.3 ± 0.6	1.8 ± 0.7	3.4 ± 1.2	3.9 ± 1.1	5.2 ± 1.2	5.3 ± 1.4
Fat-free mass (g)	2292 ± 358	2811 ± 395	3494 ± 481	3911 ± 456	4362 ± 663	4646 ± 532
Fat-free mass index (kg/m^2^)	10.0 ± 0.9	10.7 ± 0.8	10.9 ± 1.2	11.2 ± 0.9	11.1 ± 1.5	11.0 ± 1.0
**Indirect calorimetry**						
VO_2_ (mL/min)	19.9 ± 3.0	23.1 ± 4.0	41.5 ± 5.6	47.1 ± 9.1	52.9 ± 6.2	56.2 ± 8.6
VO_2_ /fat-free mass (mL/min/kg)	5.70	8.22	11.88	12.04	12.13	12.09
VCO_2_ (mL/min)	17.0 ± 3.2	18.6 ± 3.4	35.8 ± 5.6	40.1 ± 8.5	43.1 ± 5.0	46.4 ± 7.8
Respiratory Quotient	0.85 ± 0.07	0.80 ± 0.05	0.86 ± 0.04	0.85 ± 0.06	0.81 ± 0.03	0.82 ± 0.04
Energy expenditure (kcal/d)	139 ± 22	159 ± 28	287 ± 40	325 ± 64	362 ± 42	386 ± 61
Energy expenditure per fat-free mass (kcal/kg/d)	61.3 ± 10.6	56.6 ± 6.5	82.6 ± 9.0	83.1 ± 15.7	84.3 ± 9.1	84.3 ± 12.0
**Hormones**						
n	11	81	11	33	8	22
IGF-1 (ng/mL)	43 ± 21	28 ± 16	76 ± 14	59 ± 16	84 ± 28	56 ± 15
IGF-2 (ng/mL)	198 ± 56	189 ± 48	277 ± 42	272 ± 69	333 ± 71	319 ± 84
IGFBP2 (ng/mL)	1173 ± 587	1244 ± 290	765 ± 182	805 ± 211	625 ± 148	669 ± 182
IGFBP3 (ng/mL)	1434 ± 526	1325 ± 423	2252 ± 307	2200 ± 406	2891 ± 409	2538 ± 464
Leptin (µg/mL)	1.6 ± 0.8	1.3 ± 0.6	3.7 ± 2.5	3.6 ± 2.2	4.8 ± 2.2	3.3 ± 1.1
Insulin (pmol/L)	11.5 ± 18.0	3.1 ± 3.4	10.5 ± 13.5	11.6 ± 17.9	9.5 ± 8.5	12.4 ± 12.7
Cortisol (nmol/L)	22 ± 19	20 ± 27	11 ± 6	18 ± 21	22 ± 12	19 ± 20
**Biomarkers of metabolism**						
Albumin (mmol/L)	35.3 ± 3.6	36.3 ± 2.9	37.4 ± 2.9	39.5 ± 2.9	40.5 ± 2.5	42.9 ± 2.8
Protein (mmol/L)	52.6 ± 4.6	55.7 ± 4.1	51.8 ± 3.4	54.3 ± 3.6	57.7 ± 2.7	57.9 ± 3.4
Triglycerides (mmol/L)	1.4 ± 0.4	1.8 ± 0.5	1.7 ± 0.9	1.8 ± 1.0	1.6 ± 0.4	2.1 ± 0.9
Cholesterol (mmol/L)	2.8 ± 0.7	2.6 ± 0.6	3.2 ± 1.1	3.7 ± 0.6	4.1 ± 0.7	4.0 ± 0.8

Values present the mean ± standard deviation; *IGF* insulin-like growth factor, *IGFBP* insulin-like growth factor binding protein, *VCO*_*2*_ carbon dioxide production, *VO*_*2*_ oxygen consumption

Body composition data (i.e. fat mass, percent fat mass, and fat-free mass) showed similar trends in both groups from t_1_ to t_3_ (Table 2). Term-born infants typically had greater absolute fat-free mass and fat mass measurements at all time points. When fat-free mass is presented based on postmenstrual age, there was no difference between late preterm and term infants (Fig. 3B). However, trajectories for percent fat mass and fat mass were at a higher level in preterm infants (Fig. 3B). The calculated fat mass index was comparable in both groups and continuously increased from t_1_ to t_3_. The fat-free mass index levels were also comparable across groups and remained consistent at all measured time points (Table 2).

### Energy expenditure

Indirect calorimetry data showed analogous trends in both the late preterm and term-born groups. While the absolute energy expenditure increased with postnatal age in both groups, it tended to be greater in term-born infants than in late preterm infants at all time points. When calculated for energy expenditure per fat-free mass, late preterm infants showed similar values to their term-born counterparts. Respiratory quotients were 0.80 to 0.86 at all time points (Table 2).

### Growth hormones

Late preterm infants tended to show higher levels of IGF-1, IGF-2, and IGFBP-3 compared to term infants, but lower levels of IGFBP-2 (Table 2). Furthermore, IGF-1 levels tended to rise from t_1_ to t_2_ and subsequently plateaued at around 52 to 60 weeks postmenstrual age while IGF-2 and IGFBP-3 continuously increased over time. Overlapping CIs for these measurements demonstrate that the differences between preterm- and term-born levels of IGF-1, IGF-2, and IGFBP-3 are not significant. Levels of IGFBP-2 were comparable in both groups and continuously decreased throughout the study. Leptin levels were comparable in the late preterm and term-born group and tended to increase over time. Conversely, measurements of insulin differed in both groups. Insulin in the term-born group increased from t_1_ to t_2_ and subsequently plateaued, while insulin levels in the late preterm group did not show changes. Additionally, CIs overlapped for insulin measurements, rendering the difference insignificant (Table 2).

### Metabolic biomarkers and stress hormones

The analysis of metabolic biomarkers and stress hormones showed that cortisol and triglyceride levels did not change over time in both groups. Measurements of albumin and total protein tended to slightly increase from t_1_ to t_3_ and cholesterol levels increased in both groups. No significant differences were found in metabolic biomarker levels between the two groups (Table 2).

### Comparison of growth, body composition and hormones between preterm and term infants

The change in hormones and growth showed individual patterns for late preterm and term infants. Hormones (IGF and leptin, Fig. 3D), percent fat mass, fat mass (Fig. 3B), and body weight (Fig. 3A) began to increase postnatally, which was observed despite the four-week postmenstrual age difference.

In contrast, trajectory curves of length, head circumference (Fig. 3A), and fat-free mass (Fig. 3B) for preterm and term infants were similarly aligned along a single trend line.

### Correlation of growth, body composition, nutrition and hormones

The correlation analysis between anthropometric data, body composition measurements, and growth hormones revealed significant relationships. Weight, length, head circumference, fat mass, fat-free mass, skin-fold thickness, and energy expenditure per fat-free mass were positively correlated with IGF-1, IGF-2, IGFBP-3, and leptin (*p* < 0.01) while IGFBP-2 was negatively correlated (*p* < 0.01). When testing for association between the fat-free mass with growth hormones, the linear mixed model revealed that the fat-free mass of both infant groups on average was 3.8 g (95% CI: 0.5 to 7.0 g) and 1.2 g (95% CI: 0.02 to 2.4) higher with each incremental increase of 1 ng/mL of IGF-1 and IGF-2, respectively.

Additionally, it was found that the type of nutrition affects IGF levels in infants. When fed with breast milk, IGF-1 and IGF-2 levels tended to be Lower in comparison to formula. Analyzing the effect of nutrition type on growth hormones in a mixed linear model adjusted for postmenstrual age showed that formula milk was related to 8.8 ng/mL (95% CI: 1.5 to 16.1 ng/mL) higher IGF-1 levels compared to breast milk. IGF-2 tended to be higher by 16.9 ng/mL (95% CI: 4.8 to 38.6 ng/mL). Also, body composition was affected by the type of nutrition. Percent fat mass and fat mass tended to be Lower in breast-fed compared to formula-fed infants by 0.5% (95% CI: −2.2 to 1.2%) and 30 g (95% CI: −154 to 100 g), while fat-free mass tended to be higher by 14 g (95% CI: −132 to 160 g).

## Discussion

This study analyzed levels of hormones, metabolic parameters, growth, body composition, energy expenditure and nutrition in healthy late preterm and term infants during the first four months of life. During this time, significant changes in IGFs and leptin levels that paralleled fat mass accumulation were observed. Fat mass accrual started directly after birth, and therefore, at an earlier postmenstrual age in the cohort of late preterm compared to term infants. However, in this healthy and well-nourished population, fat-free mass developed along similar trajectories in both late preterm and term infants.

### Body composition

In our study, fat mass growth accelerated earlier in late preterm infants and along a steeper fat mass trajectory compared to term infants. This finding is consistent with recent reports suggesting that fat mass accretion is stimulated by the transition to the extrauterine environment [7, 8]. It has been hypothesized that birth itself may serve as a physiological trigger for rapid fat mass accumulation [7, 8]. Our previous findings from a systematic review of body composition measurements support this concept, showing that birth triggers a marked increase in fat mass accumulation – reaching an average of 25% within 2 to 3 months postnatally [8]. As a result, late preterm infants begin accumulating fat mass earlier in terms of postmenstrual age, while term infants serve as the normative reference group. In contrast, fat-free mass does not show an earlier acceleration initiated by earlier birth. Accordingly, late preterm and term infants follow a comparable growth trajectory in fat-free mass.

However, it has been reported that preterm infants are at risk of compromised postnatal growth [8]. This is often demonstrated by reduced fat-free mass in preterm infants at term-equivalent age when compared with healthy term infants. Despite this, our finding that late preterm infants continued to grow fat-free mass at the same trajectory when compared to term infants could be explained by the following facts: 1) infants were healthy and 2) sufficient postnatal nutrition was provided in amounts which allowed the late preterm infants to grow without delay during postnatal adaptation. Additionally, late preterm infants are only mildly premature and may not be as susceptible to postnatal growth restriction.

### Energy expenditure

In this study, we observed a postnatal increase in energy expenditure. It increased more than double during the first two months, which parallels the increase in fat-free mass. Similar results have also been described before [19]. However, from theoretical consideration of postnatal physiology, the rise in energy expenditure can be divided into two phases. During the first weeks of life, postnatal adaptation to the extra-uterine environment (thermogenesis, breathing, physical activity, metabolization of nutrition) promotes an increase in energy expenditure. At this time, the activity of metabolic tissue increased, which led to a rise in the relative energy expenditure of the fat-free mass. In the second phase, the observed increase in energy expenditure is caused by the proliferation of fat-free mass resulting in more metabolic active tissue. In this context and also confirmed in our study, it is accepted that the growth of metabolically active fat-free mass generates greater absolute values of energy expenditure while energy expenditure related to kilogram fat-free mass remains stable [20, 21]. As a note, the infant’s fat-free mass is a key determinant of how many calories they burn, as it represents the body's most metabolically active tissue [22].

### Postnatal changes of IGF

In this current study, IGF-1 plasma levels in term infants plateaued between two to four months of life while IGF-2 levels continuously increased. This trend was also shown by others [23]. In comparison to term infants, data for preterm infants showed a similar course in our study. However, levels of IGF-1 and IGF-2 were slightly higher in the preterm infant group compared to term-born infants. This is unexpected as an early removal from the uterine environment prematurely terminates the placenta-induced third-trimester increase in fetal IGF-1 and typically leads to lower levels in preterm-born infants [24–26]. Hellström et al. also showed that levels of IGF-1 in preterm infants declined during the immediate postnatal transition to the extrauterine environment, rather than mirroring the IGF-1 increase of late gestation [27]. Furthermore, multiple studies demonstrated that preterm-born infants generally have lower postnatal levels of IGFs than term-born infants [24, 26, 28]. Additionally, their IGF-1 concentrations are expected to increase at slower rates than in term-born infants [25, 26]. Possible reasons for the higher IGF levels in preterm infants could be the blood collection at a later day of life compared to term infants. Blood in the preterm-born group was on average at day 9 of life, when the immediate postnatal drop of IGF-1 has concluded. Further, the group of late preterm infants in this study had a higher portion of formula-fed infants (see paragraph below).

### Relation between IGF and source of nutrition

In this study, we observed that infants fed with formula showed higher levels of IGF-1 compared to breast-fed infants. As levels of IGF-1 correlate positively with weight and fat mass accretion, it could be concluded that formula-fed infants showed greater growth [29, 30]. This effect could possibly be explained by a different macronutrient composition. Recent studies have expanded upon the interrelation of nutrition and IGFs [31]. They demonstrate that hormonal responses can be modified by nutritional intake, highlighting the importance of nutrition in growth. Furthermore, the nutritional impact on growth is especially apparent in newborns, as the postnatal IGF release is nutritionally regulated via the insulin response until the growth hormone axis is fully matured at 6 to 9 months of life [27, 32]. At this time, growth hormone takes over the regulation of IGFs which results in a slight reduction of the nutritional effect on growth [29].

### Correlation between hormones and body composition

The change in leptin levels were paralleled by percent fat mass. Our observation confirms recently published data that leptin, which is released by adipocytes, can act as a marker for the quantity of adipose tissue [33, 34]. Also, leptin does signal sufficient nutrient availability, which increases the metabolic rate, thus promoting growth as shown in our study [35].

Further, our study shows an association between IGFs and fat-free mass. IGF-1 and IGF-2 levels were positively correlated with fat-free mass and increased in parallel with fat-free mass accretion. This finding reflects the recently described physiology that IGFs are mitogenic and proliferative, stimulate glucose uptake and protein synthesis, and inhibit apoptosis [25, 36]. In summary, the postnatal change of body composition and observed relation of hormones (IGF, leptin) align with physiological expectations during early growth. These findings lend support to our initial hypothesis, suggesting that the examined cohort underwent typical developmental progression.

### Postnatal change of body composition during postnatal withdrawal of maternal and placental hormones in late preterm and term infants

In our study, fat-free mass developed in late preterm infants at the same trajectory compared with term infants. However, the fat mass accelerated immediately after birth in late preterm infants. Commonly, it is described that preterm infants show postnatal growth restriction with reduced fat-free mass and higher percentage fat mass compared with term infants. This clinical course could be expected in preterm infants due to two factors. First, the nutritional intake during postnatal adaptation is often reduced which leads to growth deficits when compared to fetal counterparts. In particular, the growth of fat-free mass is interrupted by nutritional deficiency. Second, the withdrawal from the maternal–fetal unit leads to metabolic and hormonal disruption [9]. Due to a lack of growth-promoting hormones (placental growth hormone, IGF-2), the accretion of fat-free mass is repressed. Furthermore, energy-dense nutrition achieved by the end of the first week of life in combination with a relative hormonal deficiency can promote fat mass accretion. However, this fat mass accretion was described to be a physiological adaptation to the extra-uterine environment since fat mass accumulation is needed for energy storage, thermoregulation, and the subcutaneous fat mass barrier that protects against infectious diseases [37, 38].

In summarizing this finding, we observed that late preterm infants adapt to the postnatal environment in a similar course when comparing with term infants. The growth of fat-free mass is not interrupted. This indicates that the observed cohort of preterm infants developed normally.

This finding, however, has implications for the expected growth trajectory in healthy preterm infants. While the fat-free mass grows along a similar trajectory compared with term infants, fat mass growth is accelerated during the first days of life as a process of the postnatal adaptation. Therefore, during the first months of life, a higher weight compared to term infants would be expected postnatally at earlier postmenstrual age [39]. However, this suggestion contrasts the current recommendation of the American Academy of Pediatrics that preterm infants should grow similarly to their term-born counterparts. But in this study, late preterm infants had a higher weight during the first months of life corrected age than term-born infants. In addition, our earlier review confirmed the different pattern of fat mass accretion in preterm infants with a term infant’s fat mass catching up to 25% at 52 weeks postmenstrual age [8]. Differences between preterm and term-born infants diminished within three to four months of life. Our findings have to be confirmed in a larger study and may support a change in growth targets for preterm infants aiming higher weights from term-equivalent to two to three months corrected age when compared with term infants.

### Strengths and limitations

Strengths of this study include the encompassing and unique set of parameters: hormonal, metabolic, energy expenditure, and body compositional data. This variety in parameters captures the multifactorial interplay of physiological processes during postnatal adaptation and early life. The study focused on a healthy cohort of late preterm and term infants, allowing us to study normal physiology.

Our study has some limitations. Firstly, time points for measurements in preterm and term-born infants were matched by chronological age, which may obscure maturity-related differences. Additional measurements would be desirable to account for postmenstrual age since there was a difference of four weeks in gestation. Secondly, data on absolute macronutrients and feeding volume were unavailable. Furthermore, the type of feeding was not similarly distributed in the two groups. The percentage of formula-fed infants – associated with different IGF level and growth [40] – was considerably greater in the group of late preterm infants than in the term-born infants. In this study, venous samples at the age of 2 and 4 month were obtained pre-prandially (fasting), minimizing post-prandial effects on insulin and triglycerides. However, time of day was not standardized, which may introduce variability for analytes with circadian rhythms—particularly cortisol (morning peak) and leptin (nocturnal rise)—whereas total IGF-1, IGF-2, and IGFBP-3 are comparatively stable [41, 42]. The total number of late preterm infants was also smaller than the number of term infants. Lastly, a loss of follow-up occurred which may have impacted the results of the study.

## Conclusion

The shifts in postnatal hormonal profiles appear to enhance the accumulation of fat mass. This finding suggests that the postnatal environment, along with hormonal changes, plays a pivotal role in facilitating fat mass buildup. The increased percent fat mass in late preterm infants during the first months of life indicates that growth targets for preterm infants might need reconsideration to be higher when compared to term infants. However, the observed hormonal and nutritional interactions need further studies in preterm-born infants at different postmenstrual ages and levels of maturity.

## Data Availability

No datasets were generated or analysed during the current study.

## Abbreviations

A: Anthropometrical measurements
B: Blood sampling
CE: Clinical examination
CI: Confidence interval
D: Dual-energy X-ray absorptiometry
IC: Indirect calorimetry
IGF: Insulin-like growth factor
IGFBP: Insulin-like growth factor binding protein
VCO_2_: Carbon dioxide production
VO_2_: Oxygen uptake
